# Effects of Whaling on the Structure of the Southern Ocean Food Web: Insights on the “Krill Surplus” from Ecosystem Modelling

**DOI:** 10.1371/journal.pone.0114978

**Published:** 2014-12-17

**Authors:** Szymon Surma, Evgeny A. Pakhomov, Tony J. Pitcher

**Affiliations:** 1 Fisheries Centre, University of British Columbia, Vancouver, BC, Canada; 2 Department of Earth, Ocean and Atmospheric Sciences, University of British Columbia, Vancouver, BC, Canada; UC Santa Cruz Department of Ecology and Evolutionary Biology, United States of America

## Abstract

The aim of this study was to examine the ecological plausibility of the “krill surplus” hypothesis and the effects of whaling on the Southern Ocean food web using mass-balance ecosystem modelling. The depletion trajectory and unexploited biomass of each rorqual population in the Antarctic was reconstructed using yearly catch records and a set of species-specific surplus production models. The resulting estimates of the unexploited biomass of Antarctic rorquals were used to construct an Ecopath model of the Southern Ocean food web existing in 1900. The rorqual depletion trajectory was then used in an Ecosim scenario to drive rorqual biomasses and examine the “krill surplus” phenomenon and whaling effects on the food web in the years 1900–2008. An additional suite of Ecosim scenarios reflecting several hypothetical trends in Southern Ocean primary productivity were employed to examine the effect of bottom-up forcing on the documented krill biomass trend. The output of the Ecosim scenarios indicated that while the “krill surplus” hypothesis is a plausible explanation of the biomass trends observed in some penguin and pinniped species in the mid-20^th^ century, the excess krill biomass was most likely eliminated by a rapid decline in primary productivity in the years 1975–1995. Our findings suggest that changes in physical conditions in the Southern Ocean during this time period could have eliminated the ecological effects of rorqual depletion, although the mechanism responsible is currently unknown. Furthermore, a decline in iron bioavailability due to rorqual depletion may have contributed to the rapid decline in overall Southern Ocean productivity during the last quarter of the 20^th^ century. The results of this study underscore the need for further research on historical changes in the roles of top-down and bottom-up forcing in structuring the Southern Ocean food web.

## Introduction

The five species of rorquals (blue whale *Balaenoptera musculus*, fin whale *B. physalus*, sei whale *B. borealis*, southern minke whale *B. bonaerensis*, and humpback whale *Megaptera novaeangliae*) found in the Southern Ocean all feed almost exclusively on Antarctic krill [1]. By virtue of their large individual masses, mammalian metabolic rates, and unique feeding technique, these whales are capable of consuming enormous quantities of prey [2]. This factor, along with the high abundances of rorquals in the Southern Ocean prior to the onset of commercial whaling, implies that unexploited rorqual populations likely consumed a large proportion of the annual production of Antarctic krill [1], [3], [4]. While the predominant direction of the trophic interaction between rorquals and krill is still unclear [1], [5], it is quite plausible that rorquals may once have exerted some level of top-down control on prey populations, both in the Southern Ocean [3], [6], [7] and elsewhere [8]. It is also possible that in some locations and ecological contexts, such effects may still occur [6], [9].

In addition, Smetacek [4] and Nicol et al. [10] hypothesized that rorquals could have played another key role in structuring the Southern Ocean ecosystem by recycling the iron contained in the bodies of their prey and dispersing this limiting nutrient into the water column, thereby fertilizing diatom blooms and enhancing primary productivity. The latter, in turn, would have led to an increased abundance of Antarctic krill and thereby reinforced the ecosystem's high carrying capacity for rorquals. According to Smetacek [4], this positive feedback loop is the most plausible explanation for the fact that the primary productivity and abundance of both krill and rorquals in ice-free areas between the Antarctic Peninsula and South Georgia were the highest in the Antarctic, as well as for the seemingly paradoxical abundance of large pelagic consumers in the unremarkably productive Southern Ocean.

Commercial whaling in the 20^th^ century nearly eradicated the large rorquals and almost halved the biomass of smaller rorquals in the Southern Ocean [1]. Recent trends in the abundances of all Antarctic rorquals are location-specific and plagued by uncertainty derived from environmental and methodological factors [1], [11]. Nevertheless, it is clear that minke whale abundance decreased slightly towards the end of the last century [11], [12] while Antarctic humpback whale populations have made a notable recovery in recent decades [1]. However, the same cannot be said of the populations of large rorquals, particularly the formerly dominant blue whales [1], [4], [11]. According to Smetacek [4] the near-eradication of large rorquals from the Southern Ocean would have caused the positive feedback loop described above to break down, leading to declines in primary productivity and krill biomass and thus impeding the recovery of rorqual populations.

In the late 1970s, Laws [3] proposed what is currently known as the “krill surplus” hypothesis. This idea was developed in an attempt to account for apparent increases in the abundances and/or reproductive rates of smaller krill predators including penguins (king *Aptenodytes patagonicus*, gentoo *Pygoscelis papua*, Adélie *P. adeliae*, and chinstrap *P. antarctica*) and pinnipeds (crabeater seals *Lobodon carcinophagus* and Antarctic fur seals *Arctocephalus gazella*) observed in various regions of the Southern Ocean in the third quarter of the 20^th^ century [3]. It stated that the removal of most of the voracious and abundant rorquals by commercial whaling led to a noticeable decrease in the predation mortality of Antarctic krill, increasing the standing biomass of this species by a quantity of up to 150 million tonnes (∼4 t/km^2^) which Laws [3] designated the “krill surplus.” For comparison, the standing biomass of Antarctic krill required to support unexploited rorqual populations in the Southern Ocean (estimated here based on the reconstructed rorqual biomass and ecosystem model described below), is 900 million tonnes, or 25 t/km^2^. (The estimate of the required krill biomass provided by Smetacek [4] is slightly lower at 600 million tonnes, or ∼17 t/km^2^.) This would imply that the “krill surplus” estimated by Laws [3] represented an increase of 17% (or 25% according to Smetacek [4]) in the total standing biomass of this species. According to Laws [3], however, this excess krill biomass was largely utilized by pinnipeds, penguins, and flying seabirds, leading to the apparent increases in their biomasses and/or reproductive rates. The result of this process appears to have been the replacement of rorquals by pinnipeds and penguins as the dominant consumers of Antarctic krill, leading to a large-scale structural reorganization of the Southern Ocean food web [3].

The “krill surplus” hypothesis was favorably received at first, but its popularity waned towards the end of the 20^th^ century. This was due partly to an increasing emphasis on physical factors and climate change in Antarctic science [4], [13], and partly to a growing recognition that while both top-down and bottom-up factors were most likely involved, currently available data on trends in the abundances of the species concerned may not be reliable enough to conclusively determine the validity of this hypothesis [4], [5], [14], [15]. Several studies [16], [17] also pointed to a lack of consistency between species and regions in the purportedly increasing biomass trends for krill-eating pinnipeds, penguins and flying seabirds, which had been usually cited as evidence of a “krill surplus.” Fraser et al. [17] have also noted the importance of intraspecific, as opposed to interspecific, competition in seabirds, including penguins. They also drew attention to the incomplete spatial and temporal niche overlap between the resident, ice-associated penguins and the migratory, pelagic rorquals, and the potential effects of this situation on the magnitude of the “krill surplus.”

However, a number of studies and reviews published since the turn of the millennium suggested that in spite of the lacunae and inconsistencies in available data, the “krill surplus” hypothesis may well be a valid explanation of at least some changes in predator biomasses occurring in the Southern Ocean before the negative effects of global climate change on Antarctic krill biomass became more prominent in the last decades of the 20^th^ century [7], [13], [18], [19], [20], [21]. Furthermore, Ainley et al. [7], [13] have drawn attention to inconsistencies in the attempts of some scientists to explain locally variable and species-specific penguin biomass trends using a global climatic forcing paradigm given that the real trends in physical factors such as sea ice extent also appear to be geographically specific [22], [23]. In any case, the near-eradication of Antarctic rorquals provides marine ecologists with an unintended experiment in the form of an opportunity to examine the effects of overharvesting large, abundant consumers on the structure of a pelagic ecosystem [12].

A mathematical population modeling study by Mori and Butterworth [24] found that top-down effects alone could explain 20^th^-century trends in the abundances of many Antarctic predators, although rorqual population growth rates had to achieve relatively high values for this to occur. The authors also encountered certain difficulties while modelling several predator species and found the biomass estimate of the “krill surplus” first made by Laws [3] to be slightly too high [24]. Finally, Ballance et al. [6] suggested that further modeling studies may well provide valuable insights into this hotly debated issue.

The aim of the present study was to examine the ecological plausibility of the “krill surplus” hypothesis and the effect of the depletion of rorqual populations on the Southern Ocean food web using an ocean-scale ecosystem model. The plausibility of this hypothesis under various scenarios of long-term changes in Southern Ocean primary productivity, and hence the relative strength of top-down versus bottom-up forcing acting on Antarctic krill biomass in the 20^th^ century, were also investigated.

## Methods

For the purposes of this study, the boundaries of the Southern Ocean were taken to be identical with those of the area falling under the mandate of the Commission for the Conservation of Antarctic Marine Living Resources (CCAMLR), i.e. located roughly on the Antarctic Convergence. The total ocean area contained within these boundaries is approximately 36 million km^2^. A dynamic mass-balance model was constructed to represent the food web operating there in 1900, i.e. before the onset of commercial whaling, using the Ecopath with Ecosim ecosystem modelling framework [25].

This model contains a total of 18 functional groups. These include one group each of primary producers and detritus, five groups of zooplankton, two of other invertebrates, three of fish, two of seabirds, and four of marine mammals. The rorquals were split into two size-based functional groups to reflect the higher original biomasses, higher current depletion levels, and slower recovery rates of the larger species [1]. For these reasons, the large rorqual group in this model included the blue, fin, and sei whales, while the humpback and southern minke whales were placed in the small rorqual group. The input Ecopath parameters for each group, except for rorqual biomass densities, were assigned based on previous models of various Southern Ocean ecosystems [26], [27], [28] and modified to satisfy the requirement of mass balance that is fundamental to Ecopath.

**Figure 1:**
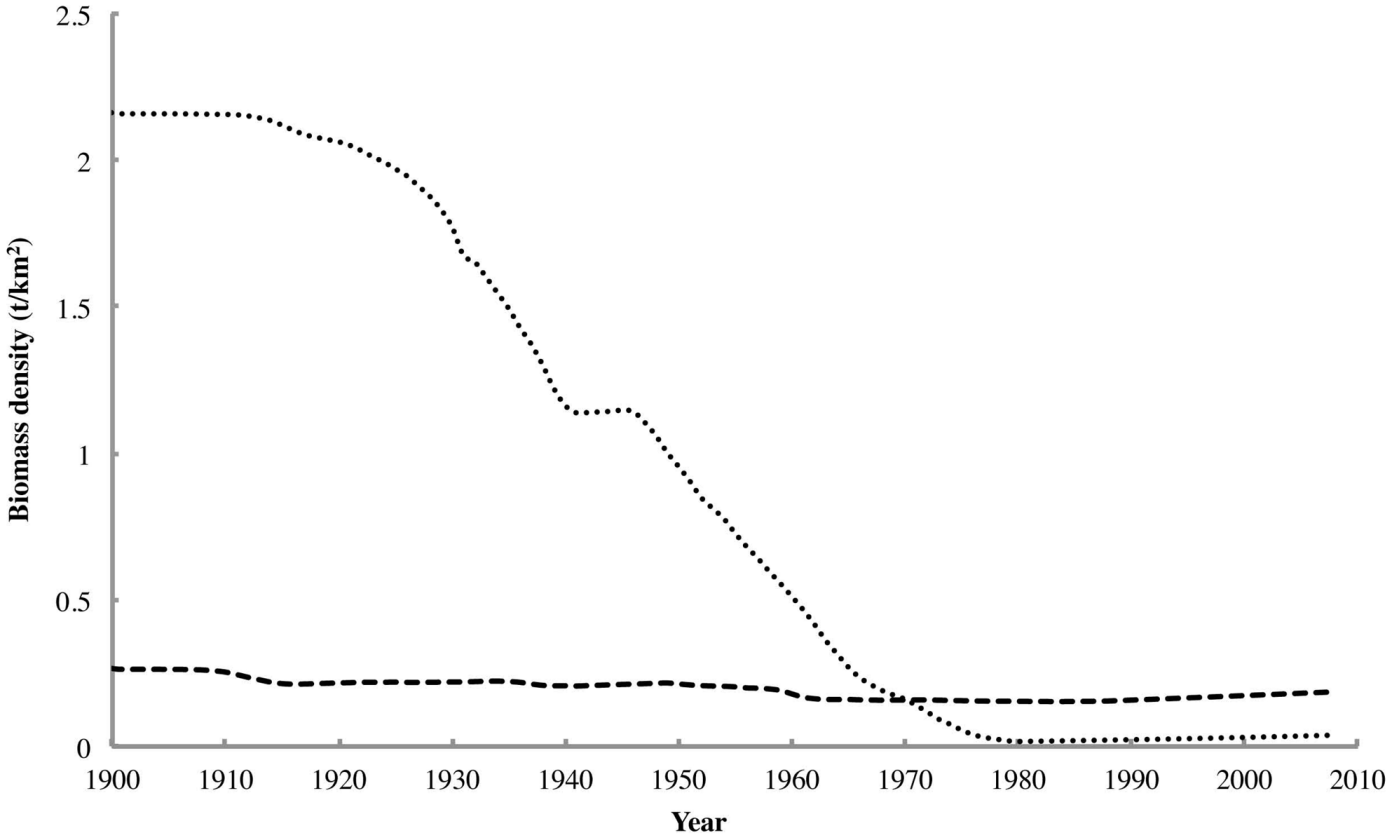
The biomass density time series for large rorquals (dotted) and small rorquals (dashed), reconstructed from IWC catch data tabulated by Leaper et al. [1]

The pre-whaling biomass densities for the two modeled rorqual groups were calculated based on the current abundance estimates and total historical catches for each whale species (the latter extracted from International Whaling Commission (IWC) records reported by Leaper et al. [1]). The current abundances and yearly catches for each species were entered into a Schaefer model to yield a reconstruction of the pre-whaling biomass (B_0_, assumed to be equal to the carrying capacity K). The estimated intrinsic rate of increase (r_c_) used in each model was based on the maximum rate of increase (r_max_) of 0.04 accepted as typical for baleen whales [29] and scaled for each species using recorded rates of increase and calving intervals. This scaling yielded values ranging from 0.05 for humpback and minke whales through 0.04 for sei whales to 0.03 for fin and 0.02 for blue whales. The yearly abundances for each species taken from the Schaefer model were converted to biomasses using the mean individual masses for each rorqual species given by Trites and Pauly [30], assuming a 1∶1 sex ratio. The reconstructed biomasses for the whale species in each functional group were summed to yield the total group biomasses. The latter were then divided by the total area of the Southern Ocean (36 million km^2^) in order to obtain pre-whaling biomass densities for entry into the Ecopath model. This process also yielded biomass time series for each rorqual group (Fig. 1), which were used to force their biomasses in the four Ecosim scenarios described below. Due to the lack of reliable time series, extending over a large fraction of the model run time, for functional groups other than rorquals, the dynamics of this model could not be validated by fitting to time series.

A sensitivity analysis of the effects of the chosen rate of increase r_c_ on the reconstructed B_0_ was conducted by applying the default value of 0.04 to each Schaefer model. This analysis yielded a 7% increase in the reconstructed pre-whaling biomass of large rorquals and a correspondingly large decrease in that of their smaller relatives. This suggests that the choice of r_c_ values has only a minor influence on the Schaefer model outputs. The effect of r_c_ on the Ecopath model appears to be even smaller, as the minimum biomass of primary producers required to support the unexploited rorqual groups decreased by only 0.8%. Furthermore, while the inclusion of all southern breeding populations of sei and humpback whales in these models may lead to a slight overestimate of B_0_ (as some of these whales feed to the north of the Antarctic Convergence [1]), the effects on the model outputs are most likely minimal. In addition, whales that were harpooned but not landed were not included in the analysis, which renders these estimates of historical abundance inherently conservative [4].

The Ecosim runs were set to begin in 1900 and end in 2008, yielding a total run time of 108 years. The start time was selected due to its significance as the year in which whalers took the first recorded rorquals in the Antarctic [1]. The runs were set to continue up to 2008 to allow sufficient time for the ecosystem to show the trophic effects of commercial whaling (which ceased after an international moratorium was declared by the IWC in 1986) and of any recovery of either rorqual group. The base Ecosim scenario employed the reconstructed biomass trajectories shown in Fig. 1 to force the biomasses of both rorqual groups, while the biomasses of all other groups were free to vary. This scenario was designed to examine the effects of rorqual depletion on the structure of the Southern Ocean food web (i.e. the “krill surplus” phenomenon) in isolation from other potential drivers of ecosystem change such as changes in primary productivity.

**Figure 2:**
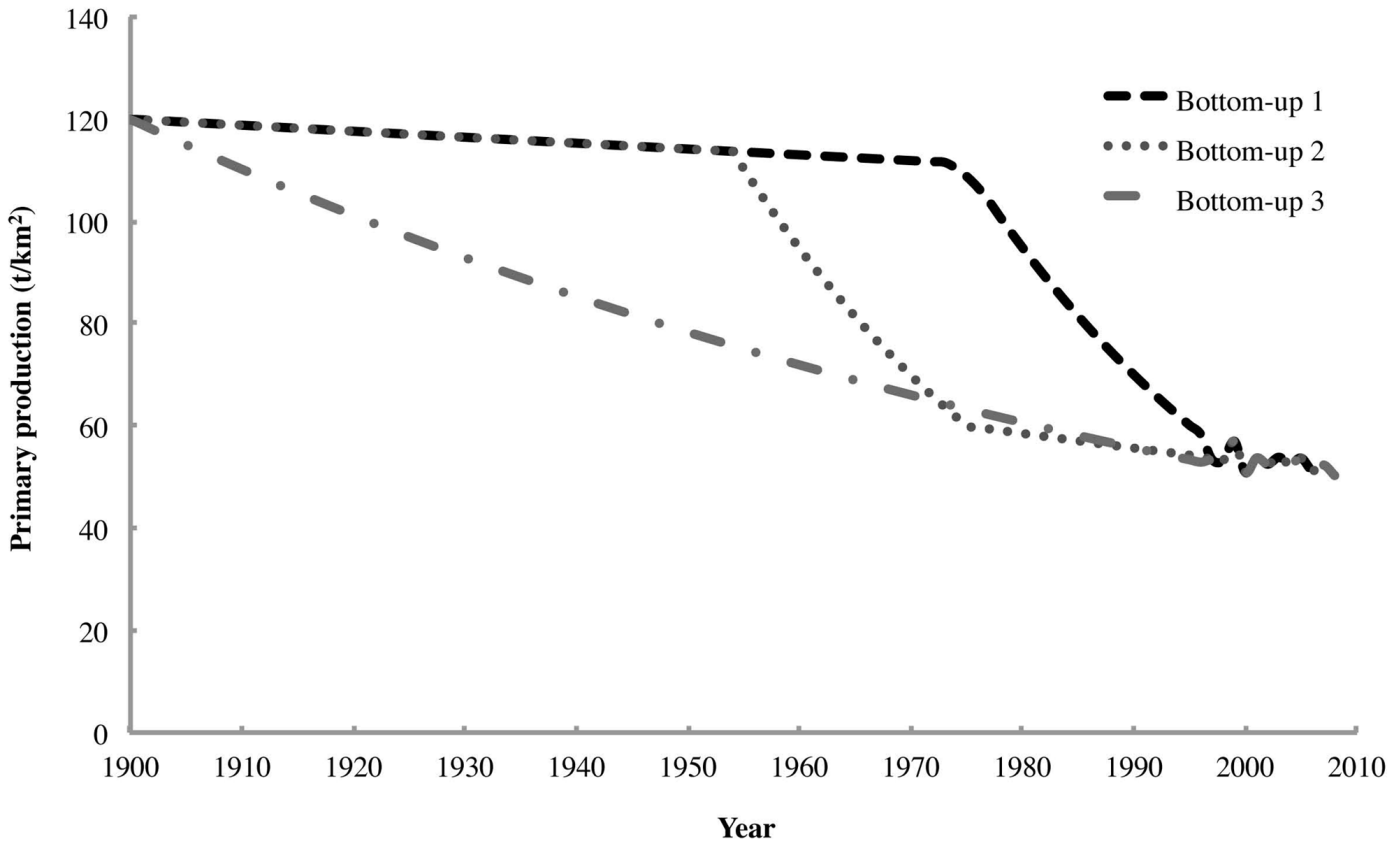
The time series for primary production in the Southern Ocean used in the three Ecosim scenarios that incorporated bottom-up forcing.

Three separate time series (Fig. 2) were used to force the biomass of primary producers in an additional set of Ecosim scenarios. These were designed to investigate the effects of bottom-up forcing on the total ecosystem response to rorqual depletion (i.e. the “krill surplus” phenomenon). More generally, they were meant to examine the potential relative strengths of top-down and bottom-up effects on the structure of the Southern Ocean food web in the 20^th^ century under various temporal patterns of primary productivity. Therefore, in each scenario, a different time series for primary producers was used in parallel with the rorqual biomass time series described above. In each of the time series, however, the starting value was derived from the primary production required by all consumers (including the unexploited rorqual populations) in 1900. The estimate of this value calculated in the Ecopath model was 120 t/km^2^. The final value for each time series was taken from the estimate of the total primary production of the Southern Ocean in 2006 obtained by Arrigo et al. [31]. Based on this later publication, the value of ∼104 t/km^2^ proposed by Arrigo et al. [32] appears to have been an overestimate. For this reason, the total modelled primary productivity was taken to have declined by 58.1% from 1900 to 2010 in all three scenarios, based on the Ecopath estimate for 1900 and Arrigo et al. [31]. This value is comparable with that of 67.4% reported by Boyce et al. [33] based on their analysis of 20^th^-century trends in Antarctic primary productivity.

The first bottom-up forcing scenario involved a gradual decrease in primary productivity lasting from 1900 until the late 1970s, followed by a phase of rapid decline that ceased in the late 1990s. This scenario was designed to simulate the observed declines in primary and secondary productivity in the Southern Ocean in the late 20^th^ century [20], [21], [34], as well as the possible delayed effects of rorqual depletion on iron availability and primary productivity in the Southern Ocean [4], [10]. Although more than half of the original rorqual biomass had already been removed by 1950 (Fig. 1), a time lag most likely occurred before the bioavailable iron in the water column had dissipated sufficiently to affect primary productivity. For this reason, it appears to be quite possible that the effects of rorqual depletion on primary productivity would not have become perceptible until the late 1970s.

The second bottom-up forcing scenario also began with a period of gradual decrease in primary productivity, but this lasted only until the late 1950s, after which a steep decline set in. This decline became more gradual once again in the late 1970s and stabilized by the late 1990s. This scenario was intended to address uncertainty in the onset of the decline in primary productivity in the Southern Ocean, particularly the possibility that this decline could have been driven by a decrease in sea ice extent which was proposed to have occurred at this time [34]. Reduced sea ice extent could have affected total primary productivity in the Southern Ocean by adversely affecting the abundance of ice algae and the size of phytoplankton blooms in the Marginal Ice Zone. The timing of the decline in primary productivity represented by this scenario could also correspond to the effects of rorqual depletion on iron availability [4], [10] if the time lag required for the bioavailable iron to dissipate is assumed to have been shorter than in the previous scenario. The third bottom-up forcing scenario entailed a gradual but still pronounced decline in primary productivity from 1900 until the stabilization period in the late 1990s, similar to the trend reported by Boyce et al. [33]. The aim of this scenario was to compare the ecological effects of a hypothetical steady decline in primary productivity to those of the more abrupt declines seen in the previous scenarios.

The sensitivity analysis described in S1 File indicates that the “krill surplus” effects observed in this study are robust to the difference between the chosen vulnerability parameter values and the Ecosim defaults (v = 2.0, Figure S1 in S1 File), but not to that between the chosen values and extreme vulnerability settings reflecting strongly bottom-up or top-down control of the food web (v = 1 Figure S2 and v = 5 Figure S3, respectively, in S1 File). In addition, the effects of rorqual depletion on many groups not involved in the “krill surplus” were sensitive to any change in the vulnerability settings. However, given that the available data [22] and past ecosystem model results [26], [27] indicate that the simulated extreme ecosystem states are unlikely to have existed in the 20^th^-century Southern Ocean, the vulnerability settings chosen in this model (Table S3 in S1 File) are unlikely to have significantly affected the results of this study regarding the ecological plausibility of the “krill surplus” hypothesis.

For further information on the Ecopath and Ecosim parameters used in this model, as well as a brief discussion of the basic tenets of the Ecopath approach, please refer to Tables S1 and S2 in S1 File.

## Results

**Figure 3:**
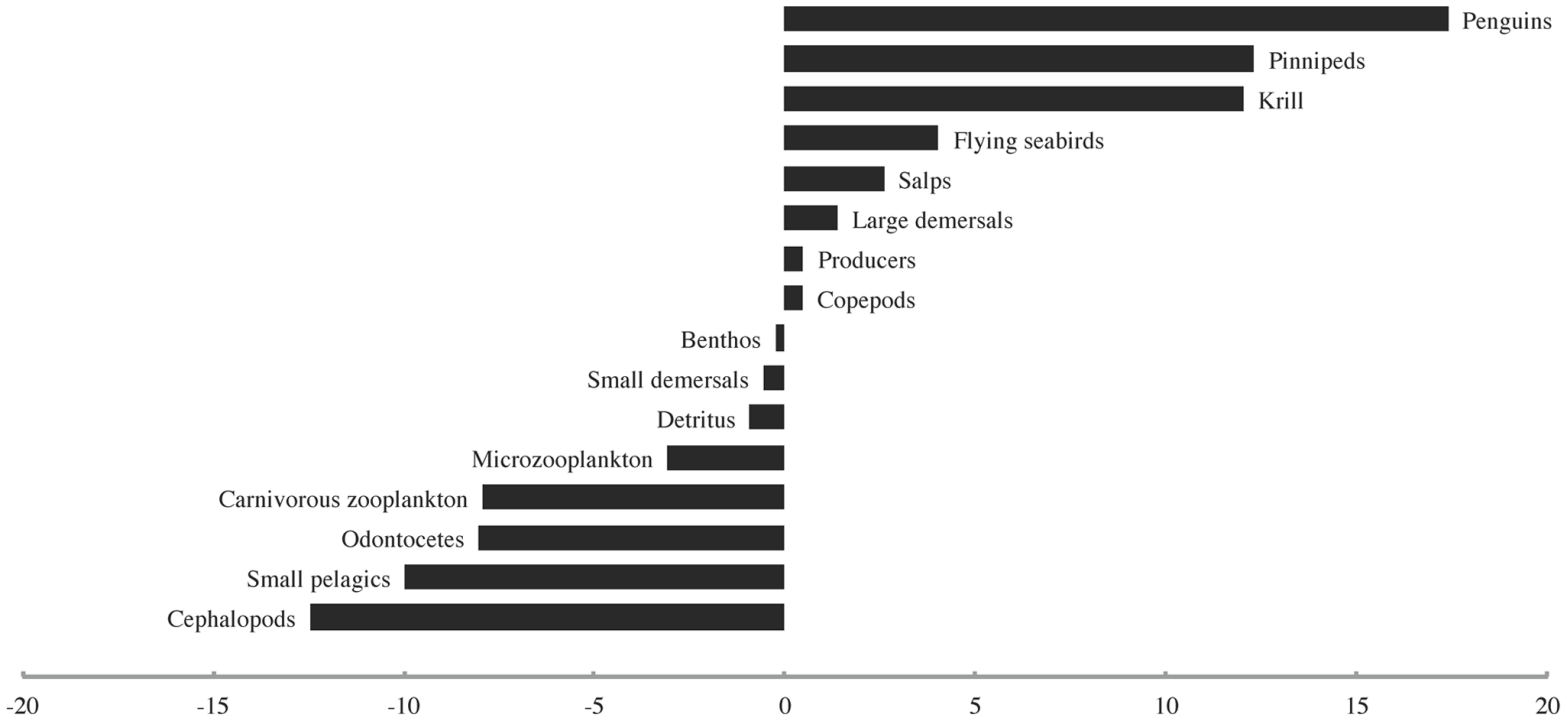
The biomass density changes (%) observed for all modelled functional groups in response to the simulated depletion of both large and small rorquals.

The simulated depletion of both large and small rorquals in the base Ecosim scenario led to marked changes in the biomasses of a number of other functional groups (Fig. 3). Four of the eighteen modelled groups registered biomass changes with absolute values in excess of 10%. The strongest increases in biomass in response to rorqual depletion were seen in penguins (∼17%), pinnipeds, and krill (∼12% each). The strongest decreases in biomass were observed for cephalopods (∼12%), small pelagics (∼10%), and odontocetes and carnivorous zooplankton (∼8% each). The “krill surplus” produced in this scenario was comparable in magnitude to the estimate proposed by Laws [3].

**Figure 4:**
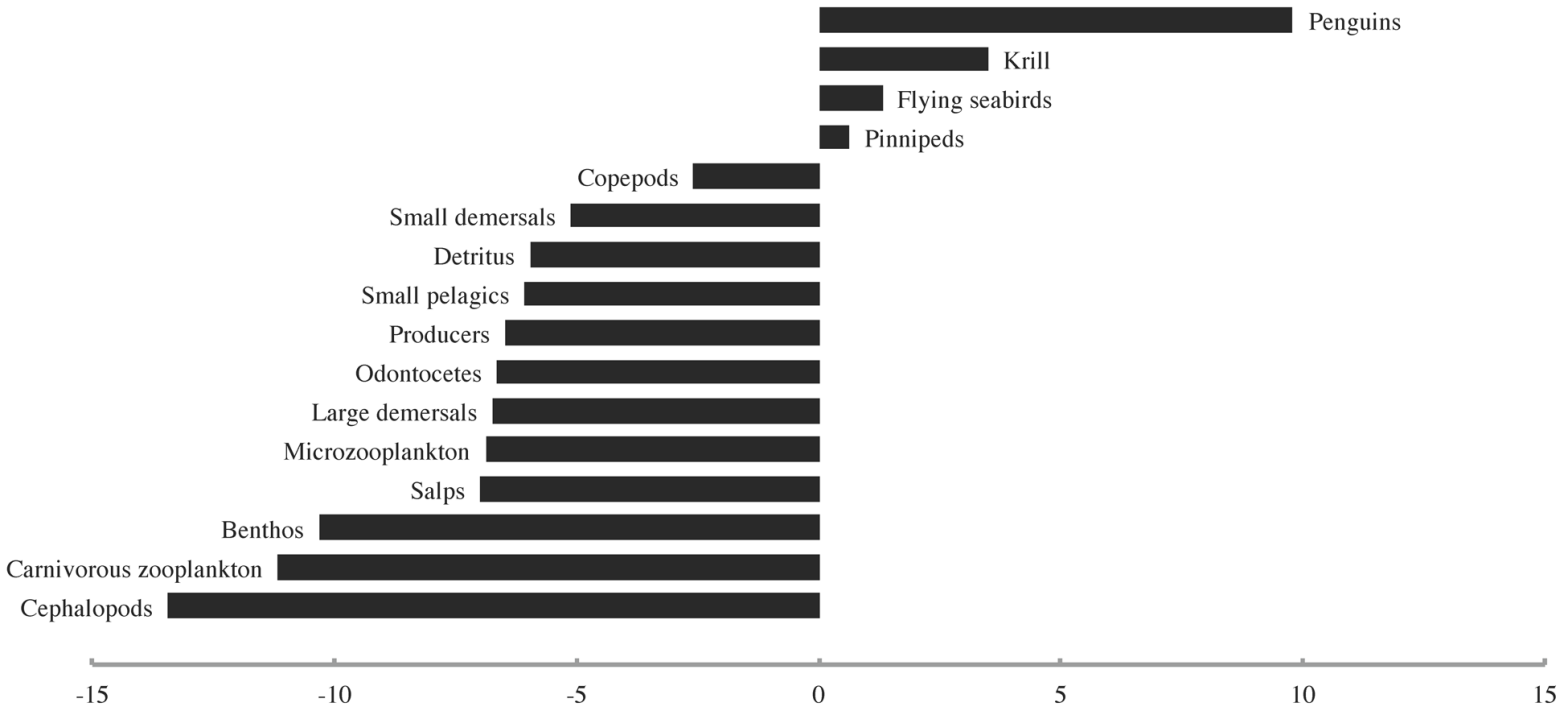
The biomass density changes (%) observed for all modelled functional groups at the peak of the simulated “krill surplus” (1976) in the first bottom-up forcing scenario.

**Figure 5:**
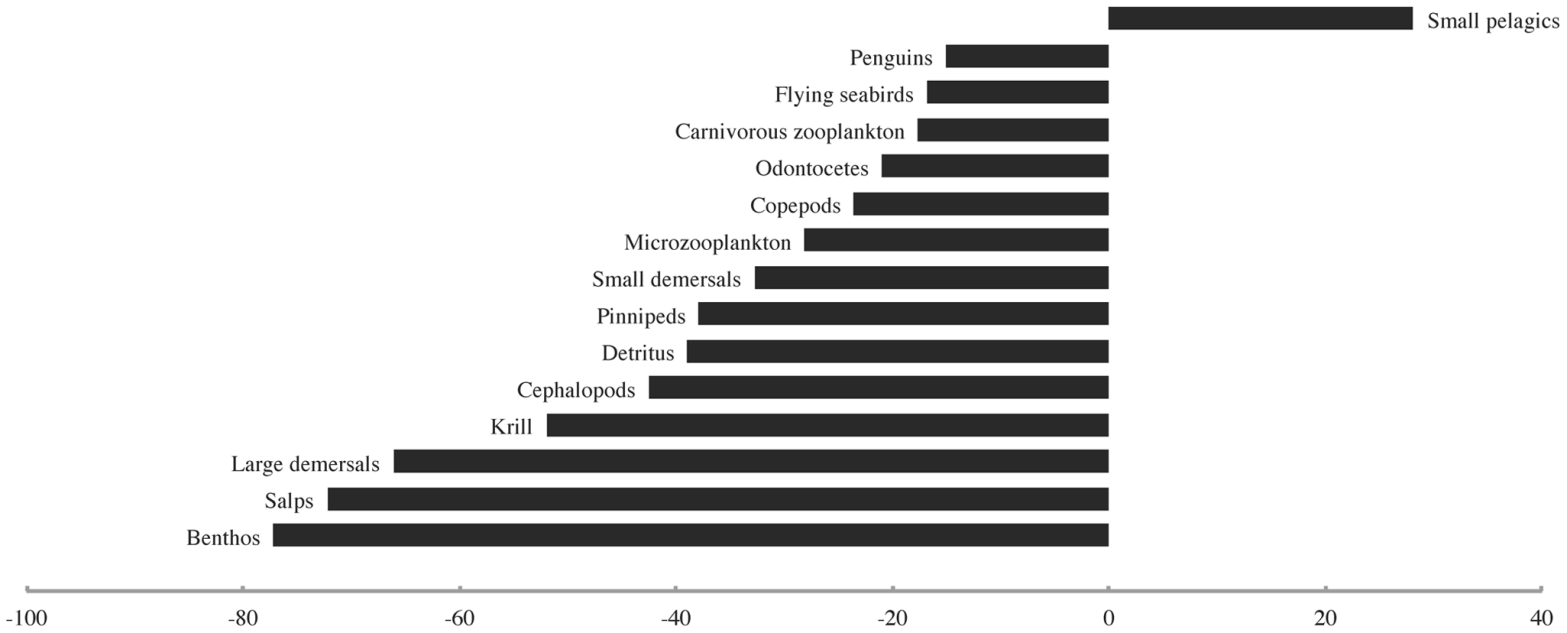
The biomass density changes (%) observed for all modelled functional groups at the end of the first bottom-up forcing scenario.

The first bottom-up forcing scenario, which involved a sharp decline in primary productivity in the last quarter of the 20^th^ century, initially yielded a “krill surplus” comparable to that postulated by Laws [3] which was later overpowered by strong bottom-up effects. By 1976, the peak year of the simulated “surplus,” the biomass of penguins had increased by ∼9.7%, that of krill by ∼3.5%, and that of flying seabirds by ∼1.3%, while most other groups had already begun to decline due to reduced primary productivity and a slight increase in predation by penguins (Fig. 4). By the end of the model run time, all groups except small pelagics (which had increased by ∼28% due to a release from predation by penguins, pinnipeds, cephalopods, etc.) had declined strongly due to a steep reduction in primary productivity (Fig. 5).

**Figure 6:**
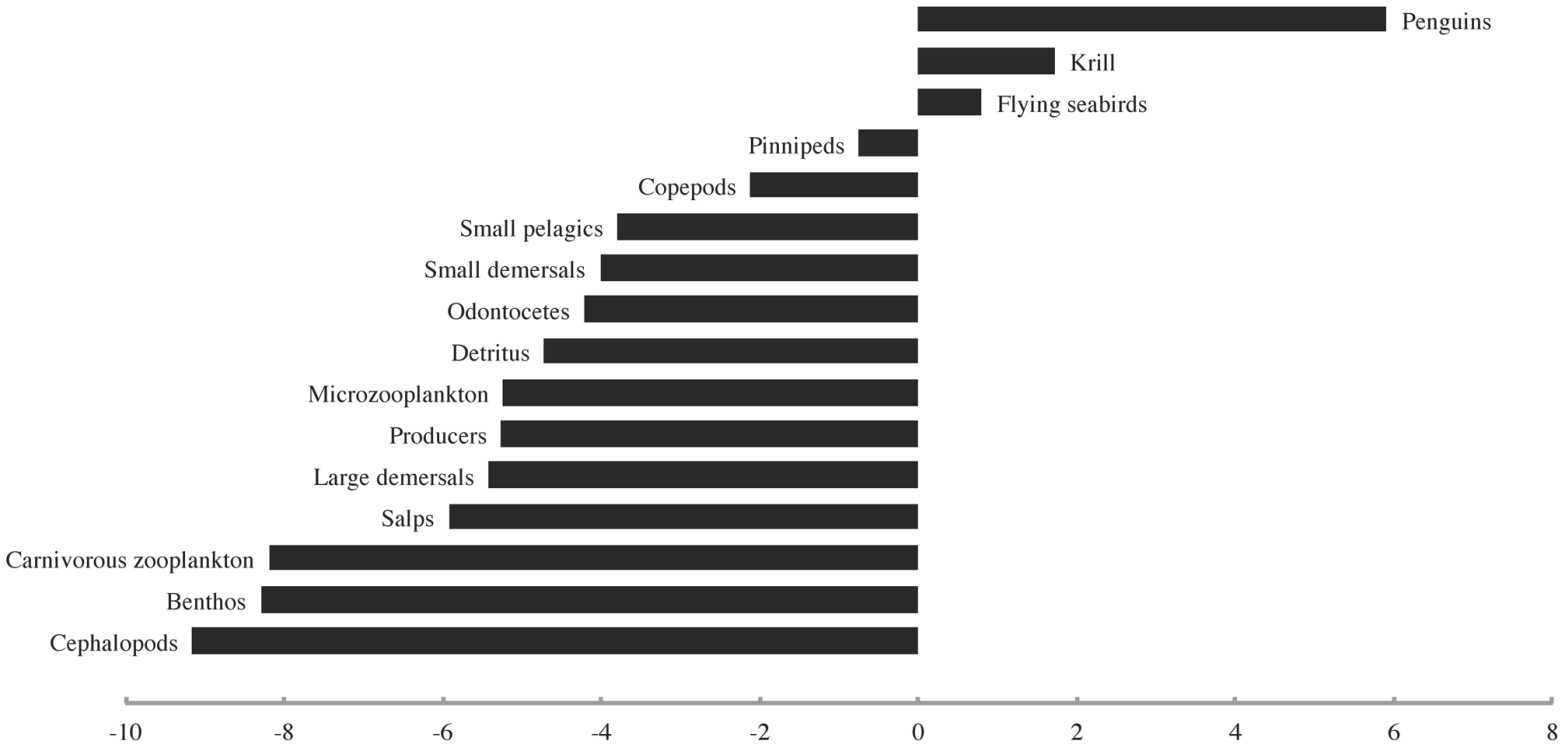
The biomass density changes (%) observed for all modelled functional groups at the peak of the simulated “krill surplus” (1954) in the second bottom-up forcing scenario.

**Figure 7:**
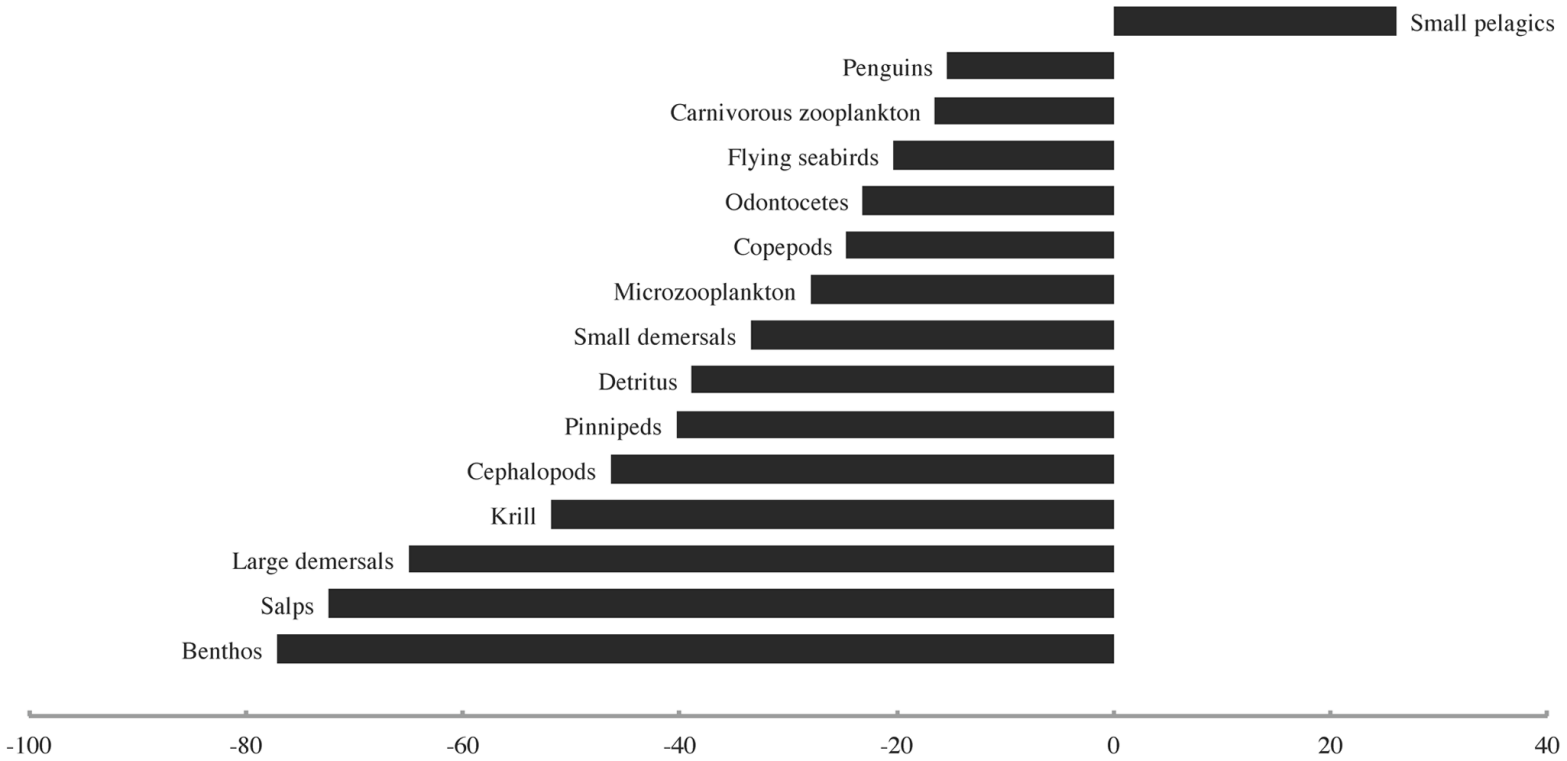
The biomass density changes (%) observed for all modeled functional groups at the end of the second bottom-up forcing scenario.

The second bottom-up forcing scenario, in which primary productivity declined sharply in the late 1950s and 1960s, produced some small and short-lived “krill surplus” effects before the onset of the decline in primary productivity. By 1954, the peak year of the “krill surplus,” the biomass density of penguins had increased by ∼5.9%, that of krill by ∼1.7%, and that of flying seabirds by ∼0.8% (Fig. 6). All other functional groups, including pinnipeds, had already registered decreases in biomass due to reduced primary productivity. By the end of the model run time, all groups except small pelagics had undergone declines which were almost identical in magnitude and rank order to those seen in the previous scenario (Fig. 7).

The third bottom-up forcing scenario, involving a gradual decline in primary productivity over the entire 20^th^ century, did not produce a “krill surplus” at any time. By the end of the model run time, all groups except small pelagics once again registered sharp declines due to reduced primary productivity. These declines were of nearly the same magnitude as in the previous scenarios, and of identical rank order as in the second one.

**Figure 8:**
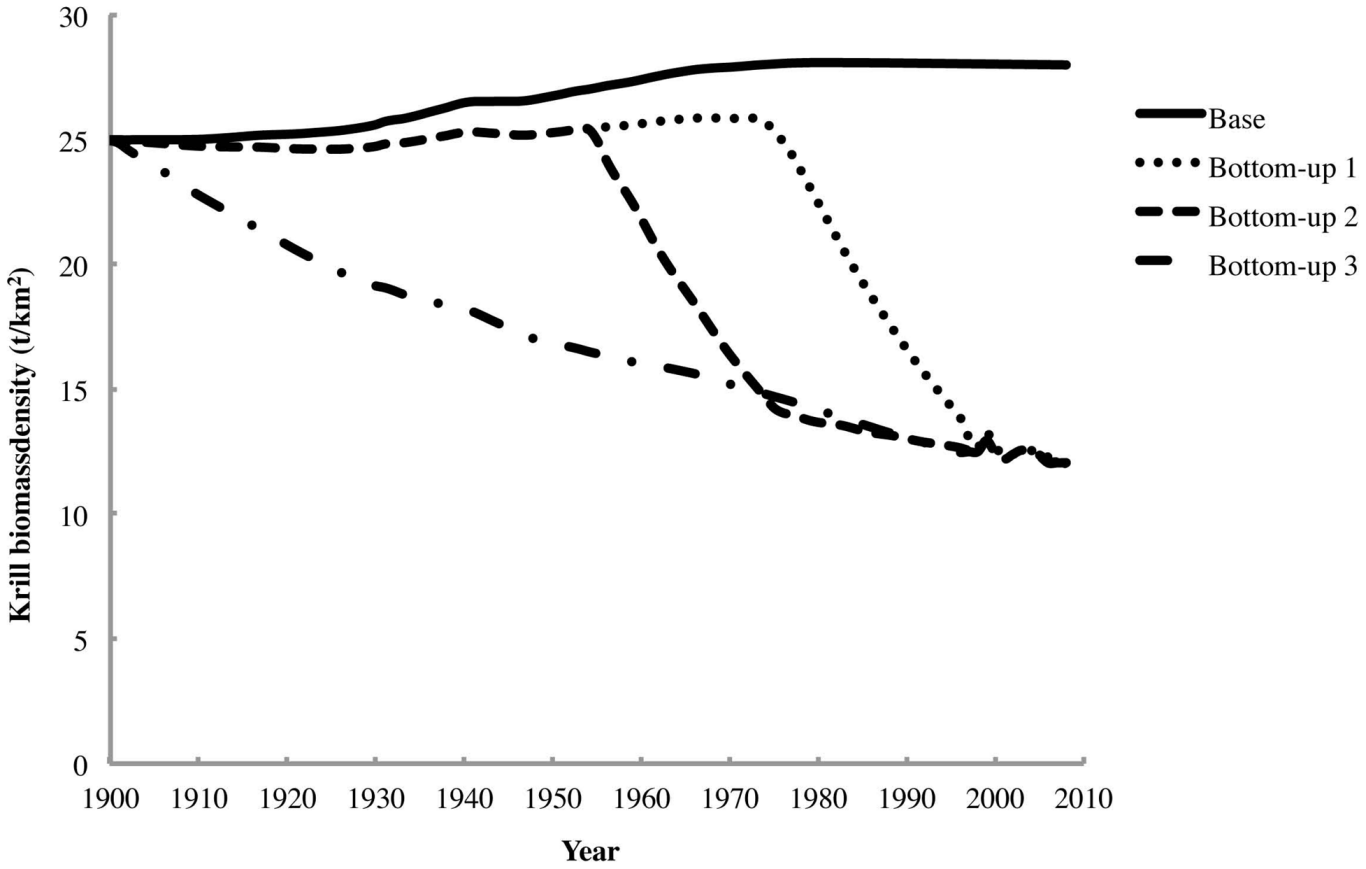
The changes in krill biomass density seen in all four Ecosim scenarios.

**Figure 9:**
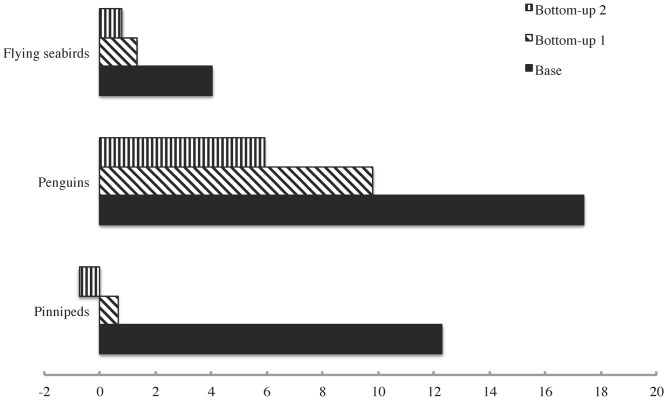
The changes in predator biomass densities coincident with the peak “krill surplus” in the first three Ecosim scenarios.

For each Ecosim scenario, the changes in krill biomass density over the model run time are shown in Fig. 8. The effects of the peak “krill surplus” on predator biomasses in the scenarios where such a surplus appeared are shown in Fig. 9.

## Discussion

The results of this study suggest that a modified version of the “krill surplus” hypothesis, along with a steep decline in primary productivity in the last quarter of the 20^th^ century that may have been caused by delayed effects of rorqual depletion, constitutes a plausible explanation of many observed changes in the structure of the Southern Ocean food web.

These results also suggest that the pre-whaling populations of rorquals in the Southern Ocean had the potential to exercise at least some level of top-down control over the biomass of Antarctic krill. This possibility is demonstrated by the marked (∼12%) increase in the biomass of this species that occurred in the base Ecosim scenario. The removal of a vast majority of the original rorqual biomass from the ecosystem appears to have released Antarctic krill from this top-down control, thereby allowing their biomass to increase. This finding is in agreement with the original “krill surplus” hypothesis formulated by Laws [3]. It is also in accordance with the conclusions reached by Ainley et al. [7], [13] regarding the viability of this hypothesis based on independent indirect measurements, as well as with the theoretical arguments of Ballance et al. [6] and Croll et al. [8] regarding the potential for top-down control of prey populations by rorquals.

However, the Ecosim simulations of combined bottom-up and top-down trophic forcing by declining rorqual and producer biomasses reveal a more complicated picture. Only the first two bottom-up forcing scenarios demonstrated increases in krill biomass at any point during the model run time, and even these were lower than the “krill surplus” postulated by Laws [3] by roughly an order of magnitude. This is most likely due to the fact that a large part of this original “krill surplus” freed by rorqual depletion was rapidly taken up by increasing populations of other predators. Furthermore, the increases in krill biomass seen in the first two bottom-up scenarios did not persist once primary productivity began to decline more steeply. In the third scenario, the effect of the gradual decline in primary productivity on krill biomass appears to have entirely overpowered that of the release from predation by the depleted rorquals. These findings suggest that relatively high and stable level of primary productivity would have been necessary to support a “krill surplus,” and that such a surplus could have been rapidly swept away by a steep decline in primary productivity, as suggested by Trathan et al. [20] and Trivelpiece et al. [21].

The high initial biomass of primary producers used as the starting value in all of the bottom-up forcing scenarios was derived from the Schaefer and Ecopath models constructed in this study, and hence is dependent on the latter's theoretical assumptions and parameter values. In addition, the true trends in primary productivity and Antarctic krill biomass in the late 20^th^ century, and the magnitude of any declines in these two variables, are still under debate, as the currently available sampling data are not entirely unequivocal. Furthermore, the biomass of Antarctic krill is inherently difficult to derive from localized field measurements of abundance due to the dynamic distribution patterns of this species [4] and the fact that most such measurements are of density rather than absolute biomass.

Nevertheless, density data are entirely compatible with the units of biomass per area (t/km^2^) required by Ecopath and Ecosim. Furthermore, if the known density values for Antarctic krill [34] were to be converted to biomass, a declining trend would still be evident, albeit one less steep than that observed for density. While the data published by Atkinson et al. [34] are admittedly the only solid source supporting a decline in krill biomass in the last quarter of the 20^th^ century, they are based on the collated results of a number of independent national monitoring programs (adding up to thousands of data points) and have been repeatedly accepted in the Antarctic literature (e.g. [20], [21]).

By contrast, the biomass data published by Reiss et al. [35], which do not suggest such a decline, are based on an acoustic survey restricted to a relatively small area surrounding the South Shetland Islands. Furthermore, they do not overlap the duration of the apparent decline but rather the period (beginning in the late 1990s) when krill biomass appears to have stabilized [36]. The estimates of krill density obtained acoustically by Brierley et al. [37] are likewise derived from a relatively small area (in this case South Georgia). Moreover, the krill population in this area is largely supported by advection rather than local reproduction. This suggests that the lack of a trend in the krill density data obtained from South Georgia may mainly reflect variable current patterns in the Scotia Sea.

While there does not appear to be any serious dispute regarding the reality of a decline in Antarctic krill biomass during the last quarter of the 20^th^ century, its magnitude is still open to debate. In satisfying the needs of mass balance and the theory underlying food web dynamics, our ecosystem model based on reconstructed historical whale biomasses and the known bioenergetic demands of large cetaceans can at least provide a robust minimum estimate of the magnitude of this decline.

The outputs of the Ecopath model discussed above suggest that a noticeably higher level of primary productivity and krill biomass existed in the Southern Ocean before the onset of rorqual depletion. These results are highly consistent with the hypothesis advanced by Smetacek [4] and Nicol et al. [10] regarding the key role of rorquals in stimulating diatom blooms via increased iron bioavailability. They indicate that the ecological reasoning behind the trends in primary productivity and krill biomass postulated by Smetacek [4] based on solid estimates of historical whale abundances and energetic requirements is quite sound. Furthermore, the results of the bottom-up forcing scenarios analyzed in Ecosim suggest that a steep decline in Antarctic primary productivity and krill biomass in the last quarter of the 20^th^ century can be reconciled with the presence of a moderate and relatively short-lived “krill surplus” during the preceding 25 years.

The results of the analysis of the sensitivity of B_0_ to r_c_ described above suggest that the known uncertainty in these parameters is unlikely to strongly affect the estimated pre-whaling biomass of primary producers in the Southern Ocean. A comparison of the pre-whaling abundance estimates obtained for each Antarctic rorqual population in this study with the lower 95% confidence limits for these quantities published by Christensen [38] (see Table S4 in S1 File) led to the same conclusion. The biomass of primary producers required to balance the Ecopath model decreased by only 0.8% in response to the use of rorqual biomass estimates based on the lower 95% confidence limits from the latter study. This outcome is not surprising given that the differences in the abundance estimates made in these two studies are quite small for the largest and historically most abundant Antarctic rorqual populations (blue and fin whales).

The historical abundance of southern blue whales as reconstructed here falls within the 95% confidence interval of the estimate obtained by Christensen [38]. While this is not true for the value published by Branch et al. [39], the use of the latter's lower 95% confidence estimates of unexploited blue whale abundance also resulted in a decrease of only 0.8% in the phytoplankton biomass needed to balance the Ecopath model. The pre-whaling biomass of Antarctic blue whales as estimated in this study (∼32 million tonnes) is also reasonably close to the value of 40 million tonnes obtained by Smetacek [4]. Thus, it appears that our estimate of the total pre-whaling primary production in the Southern Ocean is quite robust to uncertainty in Antarctic rorqual population parameters.

The results presented above also indicate that the depletion of rorquals by whaling could have had effects on other predators of Antarctic krill that correspond to the effects of the “krill surplus.” While a portion of the krill biomass no longer consumed by the rorquals may have remained free in the ecosystem as described above, the results of the base Ecosim scenario suggest that the remainder could well have fueled the increases in the biomasses and reproductive rates of some Antarctic pinnipeds and penguins noted by Laws [3]. These results are in good agreement with the conclusions reached by several researchers [7], [13], [21], [24] supporting a series of causal links between the biomasses of krill, rorquals, and competing predators. Indeed, the increase in krill biomass in the base Ecosim scenario was coincident with a ∼17% increase in the biomass of penguins and a ∼12% increase in that of pinnipeds, respectively.

However, this picture becomes a great deal more complicated when the results of the combined trophic forcing simulations are taken into account. Only the first two bottom-up forcing scenarios yielded an increase in the biomass densities of penguins and flying seabirds at any point during the model run time, and in both cases this increase was just as transient as that discussed above for krill. Furthermore, only the first of these scenarios led to any increase whatsoever in the biomass density of pinnipeds. Even the increases in penguin biomass, at values of ∼9.7% for the first and ∼5.9% for the second scenario, were considerably smaller than those seen in the base Ecosim scenario, which did not include any bottom-up forcing. These results suggest that a noticeable “krill surplus” effect on predator biomasses could have occurred even in the case of a slow decrease in primary productivity, but it would have been easily overpowered by bottom-up effects once the decline in the latter became steep. These findings agree with the conclusions of Trathan et al. [20] and Trivelpiece et al. [21], as well as those of Mori and Butterworth [24]. In addition, a “krill surplus” effect would not occur at all if primary productivity declined gradually over the entire 20^th^ century as suggested by Boyce et al. [33].

The lack of an effect on pinniped biomass in all but the first bottom-up forcing scenario is inconsistent with the observed recovery of depleted populations of Antarctic fur seals in the mid-to-late 20^th^ century [11], [40]. These results most likely reflect the lower production to biomass (P/B) ratio of pinnipeds compared to penguins in the Ecopath model. However, the pinniped functional group in this model is in all likelihood over-aggregated in ecological terms (it includes a krill specialist, two deep-diving macropredators, and two opportunistic species). For this reason, the results of these simulations may well underestimate the “krill surplus” effect on the crabeater seal, whose diet consists almost exclusively of krill, and perhaps also the leopard seal *Hydrurga leptonyx*, which uses its versatile dentition to consume both penguins and krill. In addition, the final decrease in penguin biomass seen in all three bottom-up forcing scenarios disagrees with the recently observed increase in the abundance of Adélie penguins reported by Lynch and LaRue [41]. The authors suggest that this increase may be largely due to improved breeding success at colonies on the Ross Sea due to a local rise in sea ice extent [41]. The latter may in turn mediate an increase in krill abundance, leading to enhanced prey availability for Adélie penguins, or simply provide this species with an increased habitat area. This situation illustrates the pitfalls of modelling abundance trends over a region as large and diverse as the Southern Ocean.

The effects of the depletion of rorquals on other functional groups within the model can largely be explained as consequences of the changes in krill, pinniped, and penguin biomasses described above. Thus, the decreases in the biomasses of small pelagics, cephalopods, and carnivorous zooplankton in the base Ecosim scenario most likely resulted from increased predation pressure from abundant pinnipeds and penguins. Unlike the rorquals, these predators include appreciable quantities of all three of these groups in their diets. The decline in carnivorous zooplankton may in turn have contributed to an increased biomass of salps through a release from predation pressure. The decrease in the biomass of odontocetes seems to have resulted from the decline in their main prey groups, namely cephalopods and small pelagics, as well as from the depletion of the rorquals, which are occasionally preyed upon by the Type A killer whale ecotype found in the Southern Ocean [11]. The decrease in the biomass of microzooplankton can in turn be explained by increased grazing by abundant Antarctic krill, which may also have contributed, through competition, to the lower biomass of copepods. The depletion of rorquals by whaling may also have led to a decreased production of detritus in the pelagic ecosystem.

The final results of all of the bottom-up forcing scenarios yielded declines in the biomass densities of all functional groups, except for small pelagics, due to the bottom-up effects of reduced primary productivity on the entire food web. The small pelagics seem to have proliferated as a result of a release from predation pressure by the declining penguins and cephalopods, which might imply the operation of some form of top-down effect in these interactions. The increased biomass of small pelagics appears to have exacerbated the declines in carnivorous zooplankton, salps, and copepods.

It is quite possible that the magnitude of many “krill surplus” effects generated by our Ecosim scenarios is too small for them to be detectable using the ocean sampling techniques available in the 20^th^ century. Changes of this magnitude (all <25%) would even be extremely difficult to resolve using currently available field methods. This is particularly true for small pelagics, cephalopods and most planktonic groups, and less so for large air-breathing predators. While the massive depletion of rorquals [1], [4], [38], the current recovery of Antarctic fur seals [40] and humpback whales [1], as well as recently observed changes in seabird numbers [21], [22], can be approached with some confidence, the existing field data on the “krill surplus” is incomplete and often contradictory [4], [5], [14], [15]. For this reason, ecosystem modelling is one of the few available tools that can shed light on these poorly known events in the historical ecology of the Southern Ocean.

It could also be argued that such a complex feedback network as the one that appears to link rorquals, other air-breathing predators, Antarctic krill and phytoplankton greatly limits the descriptive and explanatory utility of the theoretical concepts of top-down and bottom-up food web control. Nevertheless, it appears likely that the unexploited southern populations of rorquals were capable of exerting a certain degree of top-down forcing on the biomass of Antarctic krill. This in turn suggests that the “krill surplus” hypothesis is an ecologically plausible explanation of the observations discussed by Laws [3] in his seminal paper. However, any such surplus and its effects on the Southern Ocean food web appear to have been overpowered by the bottom-up effects of a steep decline in primary productivity in the last quarter of the 20^th^ century. Furthermore, unlike such a steep decline, the hypothesis of a gradual decrease in Southern Ocean primary productivity cannot explain the apparent decline in Antarctic krill during the last quarter of the 20^th^ century [34], [39]. The same applies to the uncertain but contemporaneously recorded trends in the biomasses of several penguin species in the Southern Ocean [21].

In addition, the apparent pattern of a steep decline in krill biomass in the 1980s and early 1990s followed by a period of stabilization [36] is almost identical to the trend in primary productivity simulated in the first bottom-up forcing scenario. Such a stabilization period could be related to the recent recovery of humpback whales [1] and Antarctic fur seals [40], which would have been unlikely to occur during the earlier period of apparent krill decline and could even potentially mask slight local recovery of krill stocks. In addition, some southern humpback whale subpopulations may derive part of their energy supply from forage fish occurring along their migration routes off Patagonia and western South Africa. Furthermore, Antarctic fur seals have a relatively diverse diet [40], suggesting that they may be able to weather periods of low krill abundance by consuming greater quantities of pelagic fish and squid utilizing the alternate copepod-myctophid food chain [19]. Such effects could help resolve the contradiction between the apparent decline in krill biomass and the recovery of some predator species from historical depletion.

The timing of the apparent decline in Antarctic krill biomass also raises doubts regarding the presence of a causal link between 20^th^-century trends in sea ice extent and primary productivity in the Southern Ocean as a whole. If such a link indeed existed, we would expect the “krill surplus” to have been overpowered by declining primary productivity in the third quarter of the 20^th^ century, when summer sea ice extent is known to have decreased in roughly two-thirds of the Southern Ocean [42], [43]. However, although data on Antarctic krill biomass during this period are lacking, the data on contemporaneous predator abundances presented by Laws [3] and the results of this ecosystem modelling study suggest that this is unlikely to have occurred. The timing of the “krill surplus” in the second bottom-up forcing scenario, representing this hypothesis, does not correspond to that reported by Laws [3]. The onset of an apparently steep decline in Antarctic krill biomass was not observed until the last quarter of the 20^th^ century [34], [36]. The hypothesis that this decline began in the late 1950s and lasted until the 1990s also cannot be tested directly. However, once again, a comparison of observed trends in krill predator biomasses [3] with the output of our ecosystem model does not support such a scenario. Furthermore, at the time when the suspected decline in Antarctic krill biomass first became evident [34], [36], sea ice extent was decreasing off the Antarctic Peninsula and in the Bellingshausen Sea [22] but increasing in the Ross Sea [23], as was the abundance of Adélie penguins [41]. These observations suggest the lack of a coherent impact of sea ice extent on Antarctic ecosystem structure during the last quarter of the 20^th^ century. This period also saw an increase in air and sea surface temperatures (SSTs) off the Antarctic Peninsula while primary productivity in the area appears to have declined [22] together with the biomasses of Antarctic krill and several predator species [20], [21], [34], [36]. It is worth noting here that the Atlantic sector of the Southern Ocean has always housed the great majority of the total biomass of rorquals and Antarctic krill [4].

Currently available data indicate that an unknown physical factor, perhaps related to increasing SSTs, may have lead to the obliteration of the “krill surplus” by decreasing primary productivity in the last quarter of the 20^th^ century, but the exact mechanism of this hypothetical physical forcing remains unclear. In the absence of new long-term records on 20^th^-century changes in various physical factors in the entire Southern Ocean (e.g. isotopic records taken from Antarctic ice or deep-sea sediment cores), this question must remain open. However, the results of this study suggest an alternative cause for the changes in the Southern Ocean ecosystem observed in the last quarter of the 20^th^ century [20], [21], [22], [34], [36]. The depletion of rorqual populations may have lead to a delayed reduction in primary productivity due to reduced bioavailability of iron [4], [10], leading to cascading effects on the biomasses of Antarctic krill and its predators. This decline in krill availability may have in turn inhibited the recovery of many rorqual populations, which has not materialized as expected (except in the case of humpback whales) despite local decreases in the biomasses of many competitor species. If this has indeed occurred, the recovery of the Southern Ocean food web is likely to be prolonged and problematic, as rorquals and primary producers may depend on each other to rebuild their biomasses to historical levels. This hypothesis is supported by the fact that the apparent decline in krill biomass [34], [36] was concentrated in the same region of the Southern Ocean (i.e. northeast of the Antarctic Peninsula) that was once richest in diatom blooms, krill swarms, and rorquals [4].

Future oceanographic research on the historical dynamics of iron bioavailability, uptake and deposition in the Southern Ocean, with particular emphasis on this geographical area, could shed further light on this hypothesis. Relevant data could be obtained from deep-sea sediment and ice cores collected throughout the Antarctic and examined for potential correlations with whale and krill biomass, sea ice extent, SST and other oceanographic variables. However, given that correlation does not necessarily imply causation, the results of such research could best be used as input for a new round of dynamic ecosystem simulations to examine the relative effects of climate change versus iron bioavailability on the changing structure of the Southern Ocean food web.

In conclusion, the findings of this study indicate that the effects of the depletion of large consumers such as rorquals on pelagic ecosystems such as the Southern Ocean may be more complex than previously thought. These effects could range from direct positive effects on prey and competitor biomasses via the relaxation of top-down control to indirect negative effects on primary productivity with bottom-up repercussions affecting the entire food web.

## Supporting Information

S1 File Figure S1, The biomass density changes (%) observed for all modeled functional groups in base Ecosim scenario runs with the adjusted (black) and default (hatched) vulnerability parameters. Figure S2, The biomass density changes (%) observed for all modeled functional groups in the base Ecosim scenario with all vulnerability parameters set to 1. Note the different scales of the axes in the three panels of the figure. Figure S3, The biomass density changes (%) observed for all modeled functional groups in the base Ecosim scenario with all vulnerability parameters set to 5. Table S1, Ecopath model parameters. Table S2, Ecopath model diet composition matrix. Table S3, Ecosim vulnerability matrix. Table S4, Historical biomass estimates and current depletion levels for Antarctic rorqual populations obtained in this study and that of Christensen [38].
(DOCX)
