# Supplementary material for: Effects of Whaling on the Structure of the Southern Ocean Food Web: Insights on the “Krill Surplus” from Ecosystem Modelling
Source: PLoS One. 2014 Dec 17;9(12):e114978. doi: 10.1371/journal.pone.0114978 (PMC4269391; doi:10.1371/journal.pone.0114978)
Supplement: S1 File — Figure S1, The biomass density changes (%) observed for all modeled functional groups in base Ecosim scenario runs with the adjusted (black) and default (hatched) vulnerability parameters. Figure S2, The biomass density changes (%) observed for all modeled functional groups in the base Ecosim scenario with all vulnerability parameters set to 1. Note the different scales of the axes in the three panels of the figure. Figure S3, The biomass density changes (%) observed for all modeled functional groups in the base Ecosim scenario with all vulnerability parameters set to 5. Table S1, Ecopath model parameters. Table S2, Ecopath model diet composition matrix. Table S3, Ecosim vulnerability matrix. Table S4, Historical biomass estimates and current depletion levels for Antarctic rorqual populations obtained in this study and that of Christensen [38]. (DOCX) [file pone.0114978.s001.docx]

**Effects of whaling on the structure of the Southern Ocean food web; insights on the “krill surplus” from ecosystem modelling**

Supplementary materials S1

S1. Past ecosystem models of Southern Ocean food webs

A number of ecosystem models have already been constructed using the Ecopath with Ecosim framework to examine the workings of ecosystems within the Southern Ocean. The latter include the Antarctic Peninsula (Erfan and Pitcher 2005, Cornejo-Donoso and Antezana 2008, Hoover et al. 2012), the South Georgia area (Bredesen 2003, Hill et al. 2012) and the Ross Sea (Pinkerton and Bradford-Grieve 2010). As a rule, the functional groups in these models were more aggregated taxonomically at lower trophic levels.

Erfan and Pitcher (2005) constructed a preliminary ecosystem model of the Antarctic Peninsula with the aim of evaluating the impact of the commercial krill fishery on predator populations. This model included 120 species aggregated into 39 functional groups, with krill being split into adult and juvenile groups. It also incorporated a spatial component constructed in Ecospace. Due to its preliminary nature, this model appears to have been intended mainly as a starting point for future studies. The second major ecosystem model of the Antarctic Peninsula (Cornejo-Donoso and Antezana 2008) was also described as preliminary by the authors, contained 28 functional groups, and had the aim of examining the patterns of biomass flow in this ecosystem. It yielded the image of a food web dominated by the flow of biomass from phytoplankton through krill to upper-level avian and mammalian predators (various species of penguins, baleen whales, seals, and toothed whales). The latest model of this region (Hoover et al. 2012) included a total of 58 functional groups (five of which contained life stages of krill) and was intended to recreate the past states of the Antarctic Peninsula ecosystem. A number of bottom-up forcing functions were used to test the effects of various physical factors on the entire food web. The study found that the effects of declines in sea ice cover on primary production percolated up through krill to affect species occupying higher trophic levels.

Bredesen (2003) built the first ecosystem model of the South Georgia area, containing 30 functional groups, with the goal of examining the potential effects of the krill fishery on the local food web. The model predicted that the fishery in question could cause small reductions in the abundances of several predatory species, and that bycatch could cause substantial decreases in finfish biomass. It also suggested that finfish could play a larger role in this ecosystem than has been previously recognized, and that the unique trophic importance of krill may have been slightly overstated. The model also indicated that the increase in Antarctic fur seal biomass in the second half of the 20^th^ century was most likely due to recovery from prior overexploitation rather than to a “krill surplus.” It also suggested that the recovery of whale populations in this region may well be hindered by the high abundance of certain fish, rather than penguins and pinnipeds. The latest model of this region, built by Hill et al. (2012) and containing 30 functional groups, suggested that flying seabirds and fish both accounted for high percentages of the total consumption of krill, and that reduced krill biomass is likely to have quite strong impacts on predator populations. Finally, the ecosystem model of the Ross Sea constructed by Pinkerton et al. (2010), containing 38 functional groups, was intended to investigate the effects of the longline fishery for Antarctic toothfish (*Dissostichus mawsoni*) on the local ecosystem. It indicated that while Antarctic toothfish is an important link in the food web, the fishery in question is unlikely to have major effects on predator or prey biomasses.

S2. Ecopath with Ecosim: an overview

Ecopath with Ecosim (EwE) is a software package designed for mass-balance modeling of food webs, ecosystem structure, and ecological interactions (Christensen and Walters 2004). It consists of three major components: Ecopath, Ecosim, and Ecospace, along with a number of smaller modules.

Ecopath creates a static snapshot of a food web by aggregating all of the biomass in the ecosystem (including detritus) into a series of functional groups. Each of these groups is characterized by a number of parameters, including biomass (B), production per biomass (P/B), consumption per biomass (Q/B), ecotrophic efficiency (EE), a diet composition matrix, and other quantities (Christensen and Pauly 1992). Functional groups composed of primary producers or detritus are naturally not assigned Q/B values. The known values of the parameters listed above are entered directly into the model, while the unknown values are calculated based on Ecopath’s fundamental principle of mass balance. The latter states that the biomass gains (from primary production and/or consumption and assimilation) and losses (from catch, predation, other sources of mortality, unassimilated food, and waste products) must balance for each functional group, i.e. more biomass cannot be lost than is gained. This principle is expressed in the “master equation” of Ecopath (Christensen and Pauly 1992).

Ecosim is a time-dynamic simulation tool based on the underlying logic of Ecopath. It uses a set of differential equations to project the biomasses of each modeled functional group forward in time in response to forcing from oceanographic (e.g. SST or salinity; usually used only for lower trophic levels) ecological (e.g. predator, prey, or competitor biomass), or anthropogenic (usually fishing effort) factors (Christensen and Walters 2004). Ecosim requires that vulnerability parameters based on foraging arena theory (Ahrens et al. 2012) be defined for each predator-prey interaction and used to express the degree of bottom-up versus top-down control of the latter. Ecospace is a spatial modeling tool originally designed for evaluating the ecological effects of marine protected areas (Walters et al. 1999). It examines ecological interactions in a spatially explicit manner by running a complete Ecosim simulation in each grid cell of a map. Each cell is connected to its neighbors by biomass flows. The magnitudes of these flows are determined by dispersal rates and habitat quality weighting parameters, both of which are specified for each functional group (Walters et al. 1999).

S3. Ecosystem model structure and data sources

*S3.1. Ecopath functional groups*

In the designation of the functional groups in this model, the primary consideration was an accurate representation of the trophic interactions between krill, rorquals, and the latter’s competitors, with general ecological realism and precision in modeling the entire ecosystem a close second. For these reasons, many of the groups used in this model (e.g. odontocetes, pinnipeds, flying seabirds, carnivorous zooplankton, microzooplankton, benthos, and producers) are almost certainly over-aggregated both taxonomically and/or ecologically. This is especially true from the perspective of a general-purpose, ecologically faithful model of the Southern Ocean ecosystem, but the functional groups used appear to provide sufficient resolution from the point of view of the focus on interactions relevant to the “krill surplus” hypothesis.

All species of krill found in the Southern Ocean (mainly Antarctic krill *Euphausia superba* and crystal krill *E. crystallorophias*) were aggregated in a single group due to their similar trophic roles. This was also done for the morphologically and taxonomically diverse producers (phytoplankton, ice algae, and macrophytes), carnivorous zooplankton (crustaceans, cnidarians, ctenophores, chaetognaths, etc.) microzooplankton (all non-photosynthetic single-celled eukaryotes), benthos (sponges, cnidarians, echinoderms, molluscs, crustaceans, etc.) and pelagic and demersal fish (toothfish, icefish, myctophids, etc). For reasons of simplicity and clarity, multi-stanza modeling of trophic ontogeny was not used for any of the taxa present in the Southern Ocean food web.

*S3.2 Ecopath parameters*

The Ecopath parameters that were used as input in the present model were based on those employed in the previous ecosystem models of various regions of the Southern Ocean (the Antarctic Peninsula, South Georgia, and the Ross Sea) described in section S3.1. As all of these coastal regions boast much higher productivities and biomass densities at all trophic levels than do the open, deep waters of the Southern Ocean, all of the estimated biomass densities taken from these regional models were multiplied by a correction factor of 0.2 to produce estimates for the study area as a whole. The validity of this correction factor was verified through a comparison of the estimated biomass density of large rorquals in the entire Southern Ocean (taken from estimates of total abundances by species found in Leaper et al. 2008) with the densities used in the regional models. The ratio obtained by this method was quite close to the chosen correction factor of 0.2.

In situations when a functional group used in the present model only occurred in one of the source models (e.g. carnivorous zooplankton in Hill et al. 2012), the biological parameters P/B and Q/B were taken from this prior model. In cases where the group concerned (e.g. krill) occurred in a number of source models, the value that appeared to be most biologically reasonable was selected and modified slightly upward or downward to reflect where the other values lay relative to the chosen value. In cases where the functional groups in the chosen model were disaggregated compared to the present model, the parameters used were estimated as averages of those from the finer-grained groups weighted by the latter’s biomasses. The biomass densities of the finer-scale groups were summed in order to obtain the biomass density for the aggregated group.

*S3.3. Balancing the Ecopath model*

The initial values of the Ecopath parameters obtained by the methods described above yielded a model that did not fulfill the requirement of mass balance (for 10 out of 18 functional groups, EE > 1). The model was balanced using incremental changes to the B and Q/B parameters and diet compositions of the affected groups. These changes were made in such a way as to decrease the predation mortality for prey groups until EE < 1.

*S3.4. Ecosim parameters and sensitivity analysis*

The Ecosim runs were set to begin in 1900 and end in 2008. The start time was selected due to its significance as the year in which whalers took the first recorded rorquals in the Antarctic (Leaper et al. 2008). The runs were set to last until 2008 to allow sufficient time for the ecosystem to show the trophic effects of commercial whaling (which ceased after an international moratorium was declared by the IWC in 1986) as well as any recovery of either rorqual group.

The vulnerability parameters for each trophic interaction between functional groups, whose value increases with the degree of exchange between the “pools” of prey that are vulnerable versus invulnerable to a given predator according to foraging arena theory (Ahrens et al. 2012), were adjusted upwards from their default value of 2.0 for most trophic interactions in the food web. This increase reflected a higher level of top-down control of the predator-prey interaction than that typically assumed by Ecosim. The reason for this decision lay in the relative scarcity of spatial refuges for zooplankton, cephalopods, and fish hunted by pelagic predators. These include many deep-diving seabirds (e.g. penguins, diving petrels, and shags) and marine mammals (e.g many odontocetes, the Weddell and Ross seals, and to a lesser extent other cetaceans and pinnipeds), as well as numerous and diverse fish species.

A sensitivity analysis was also conducted to examine the effects of various vulnerability settings on the biomass density changes produced by the Ecosim base scenario. Firstly, a test Ecosim run was performed with the default vulnerability parameters restored. The results of this run, when compared with those obtained using the modified vulnerability settings (Figure S1), demonstrated that the use of the adjusted vulnerability settings did not result in any notable changes in the result of the simulations for the functional groups of greatest relevance to the “krill surplus” hypothesis (i.e. krill, penguins, and pinnipeds), although some other groups (e.g. odontocetes, small pelagics, large and small demersals, and cephalopods) were quite strongly affected.


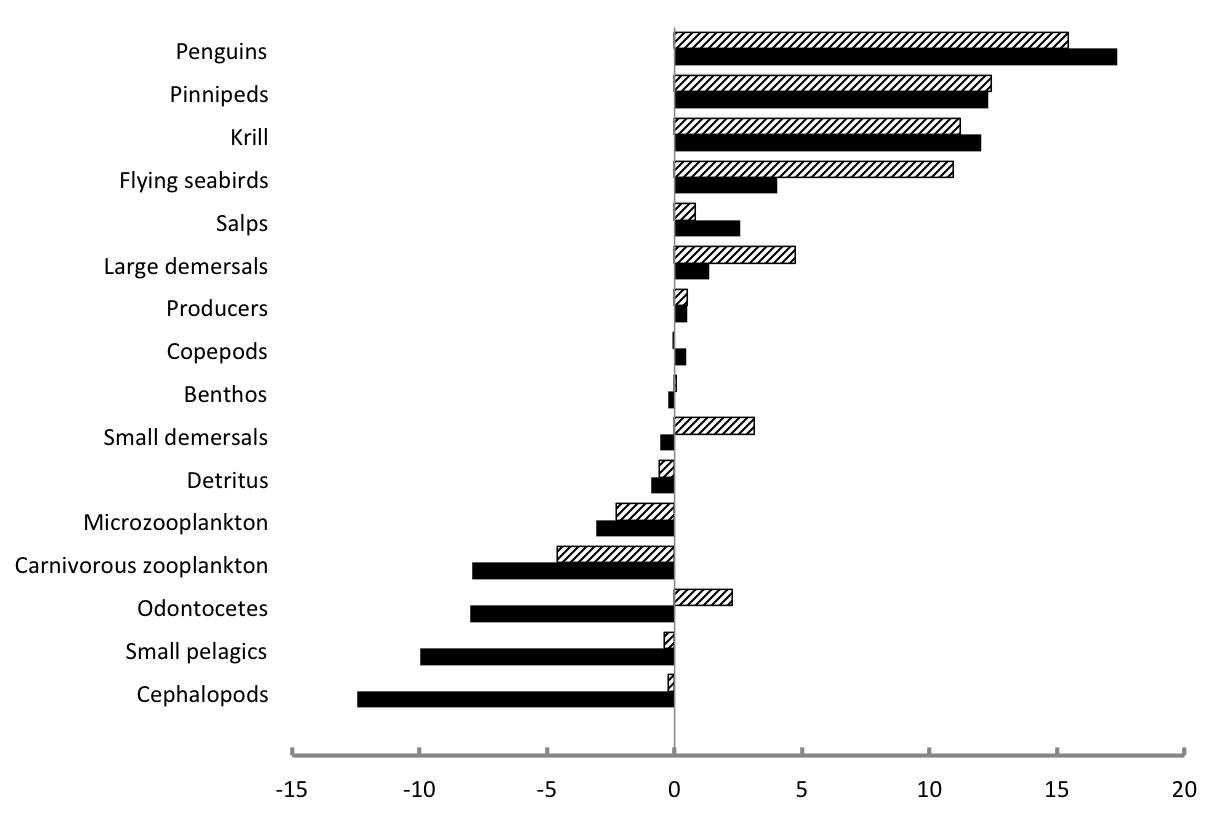


Figure S1. The biomass density changes (%) observed for all modeled functional groups in base Ecosim scenario runs with the adjusted (black) and default (hatched) vulnerability parameters.

In addition, two more Ecosim runs were performed in order to examine the effects of more extreme vulnerability settings on the results of the base scenario. In the first run, the vulnerabilities for all predator-prey interactions in the Ecopath model were set to 1.0 so as to investigate the effect of a hypothetical strongly bottom-up ecosystem structure on the output of the base scenario. The results of this run (Figure S2) indicate that, as could be predicted from basic ecological theory, the effects of rorqual depletion on such a donor-controlled Southern Ocean food web would be negative but minimal (except for odontocetes), and a “krill surplus” of any sort would not be observed.


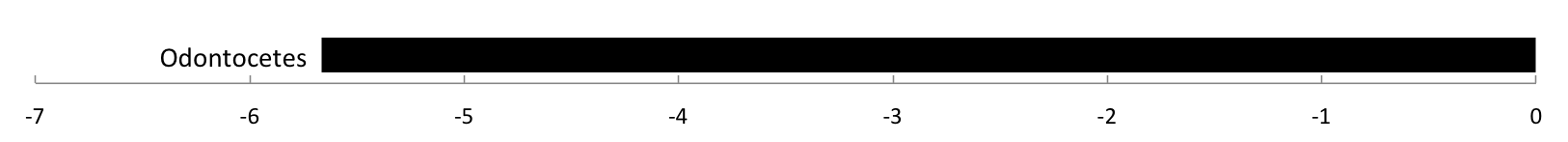


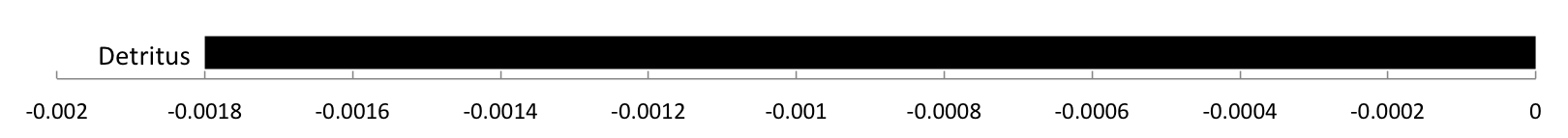


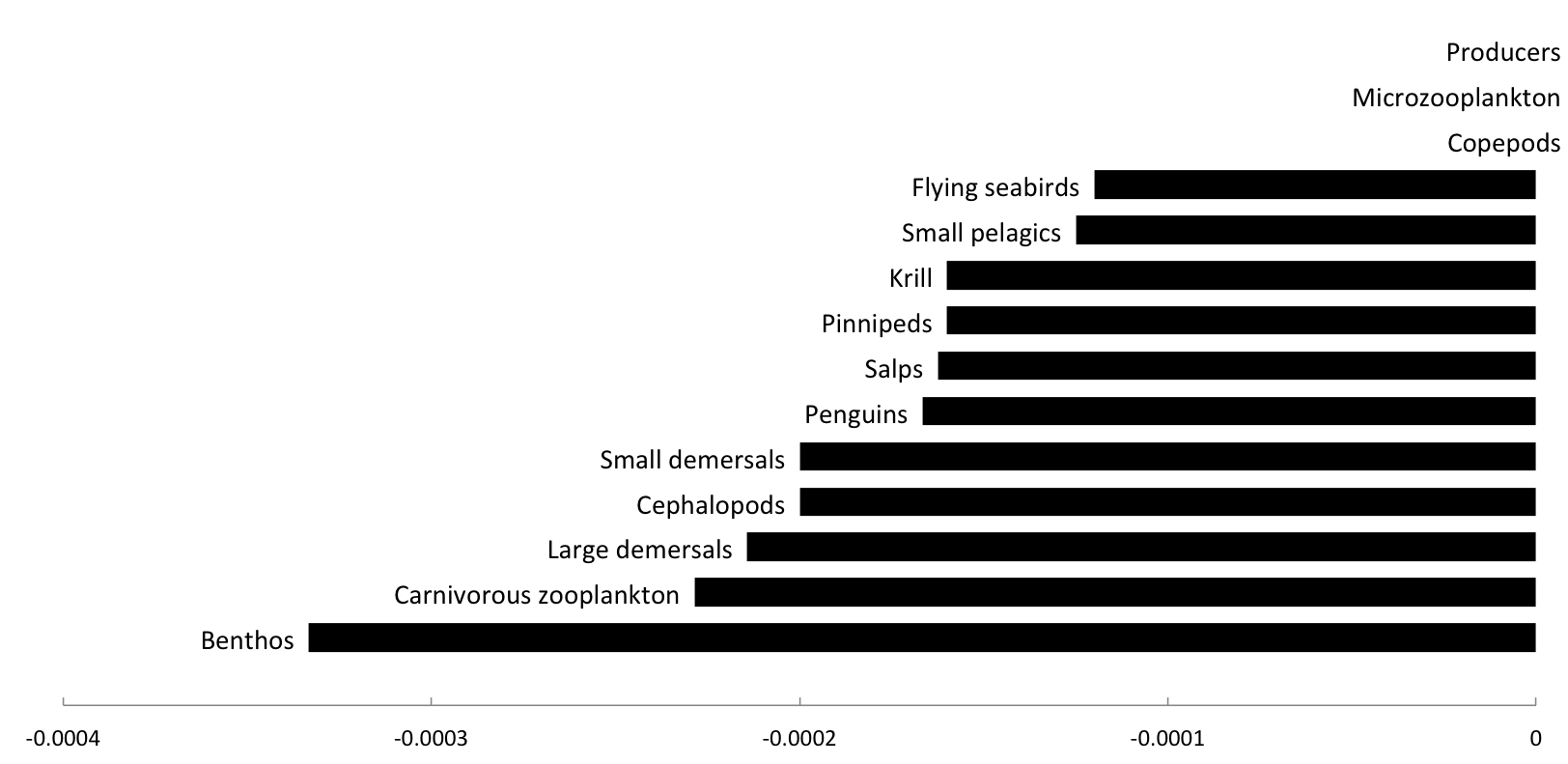


Figure S2. The biomass density changes (%) observed for all modeled functional groups in the base Ecosim scenario with all vulnerability parameters set to 1. Note the different scales of the axes in the three panels of the figure.

In the second Ecosim run, a value of 5.0 was assigned to the vulnerability settings for all predator-prey interactions in the Ecopath model in order to examine the effects of a hypothetical top-down ecosystem structure on the results of the base scenario. The results of this run (Figure S3) suggest that, as could be predicted, the effects of rorqual depletion would be exacerbated if the true state of the Southern Ocean food web included strong top-down control. In such a situation, in addition to a strong “krill surplus,” the release of primary producers from the grazing pressure of microzooplankton and copepods would lead to a strong increase in the biomass density of salps. In addition, the biomass density of small pelagics would decline precipitously as a result of increased predation pressure from abundant pinnipeds and penguins. Given that krill and salp abundance does not appear to vary synchronously (Lee et al. 2010), this state of affairs is unlikely to hold.


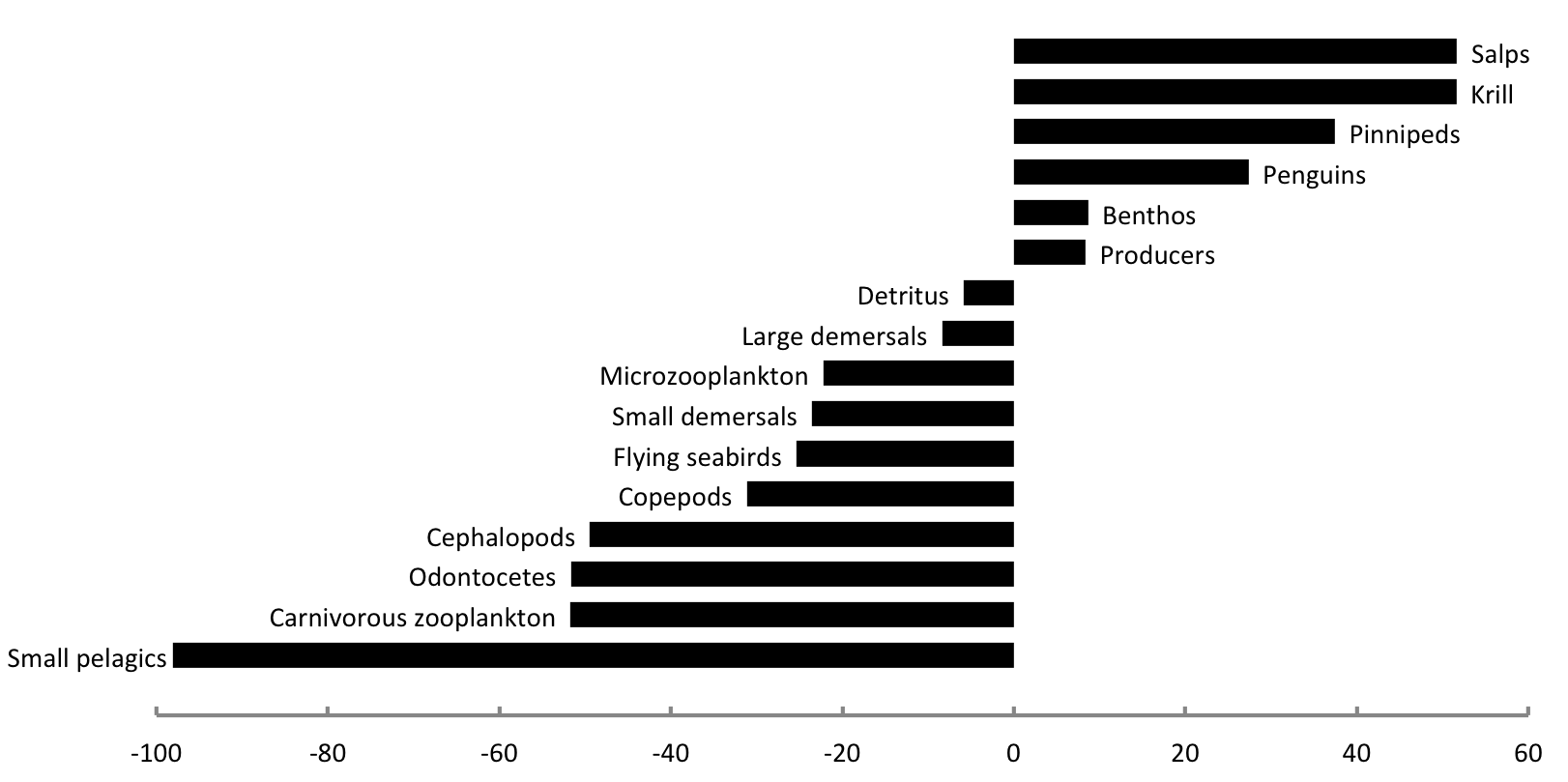


Figure S3. The biomass density changes (%) observed for all modeled functional groups in the base Ecosim scenario with all vulnerability parameters set to 5.

The results of this sensitivity analysis provide an important insight into the potential effects of errors in vulnerability estimation and possible extreme ecosystem states on the predictive power of Ecosim and its ability to accurately simulate the effects of rorqual depletion on the structure of the Southern Ocean food web. However, such extreme states do not appear very likely to have existed in the 20^th^-century Southern Ocean food web. Bredesen (2003), using finfish and krill catch data to fit her model of the South Georgia food web, generated a matrix in which most predator-prey interactions were assigned the default vulnerability value. Erfan and Pitcher (2005) scaled the vulnerability values in their model of the Antarctic Peninsula ecosystem according to the trophic level of the prey to obtain a vulnerability matrix including values ranging from 2.0 to 5.18, with 3.0 being the typical value. Hoover et al. (2012), fitting their model of the same area to time series data on krill and salp biomasses and various physical indices, generated a matrix in which most vulnerabilities remained at the default value of 2.0. The values obtained by Erfan and Pitcher (2005), derived as they are from a technique reflecting basic ecological principles rather than from time series data representing a limited selection of functional groups, are perhaps most likely to represent the true state of affairs. They are also the closest to the values used in the present model

S4. Ecopath and Ecosim model parameters

|  | Group name | TL | B (t/km²) | P/B (/year) | Q/B (/year) | EE | P/Q |
| --- | --- | --- | --- | --- | --- | --- | --- |
| 1 | Large rorquals | 3.54 | 2.16 | 0.03 | 3.75 | 0.06 | 0.01 |
| 2 | Small rorquals | 3.60 | 0.26 | 0.06 | 5.00 | 0.51 | 0.01 |
| 3 | Odontocetes | 4.88 | 0.23 | 0.06 | 7.20 | 0.60 | 0.01 |
| 4 | Pinnipeds | 4.33 | 0.25 | 0.40 | 15.00 | 0.81 | 0.03 |
| 5 | Penguins | 4.10 | 0.30 | 0.75 | 75.00 | 0.87 | 0.01 |
| 6 | Flying seabirds | 4.20 | 0.08 | 0.75 | 100.00 | 0.70 | 0.01 |
| 7 | Large demersals | 3.92 | 1.40 | 0.55 | 3.30 | 0.77 | 0.17 |
| 8 | Small demersals | 3.64 | 3.00 | 1.50 | 4.20 | 0.96 | 0.36 |
| 9 | Small pelagics | 3.68 | 8.00 | 2.00 | 5.00 | 0.98 | 0.40 |
| 10 | Cephalopods | 4.05 | 4.50 | 2.00 | 3.00 | 0.98 | 0.67 |
| 11 | Carnivorous zooplankton | 3.16 | 7.00 | 7.50 | 19.00 | 0.88 | 0.39 |
| 12 | Krill | 2.44 | 25.00 | 2.50 | 33.00 | 0.64 | 0.08 |
| 13 | Salps | 2.22 | 8.00 | 6.50 | 250.00 | 0.23 | 0.03 |
| 14 | Copepods | 2.35 | 16.00 | 15.00 | 34.00 | 0.97 | 0.44 |
| 15 | Microzooplankton | 2.00 | 40.00 | 47.00 | 160.00 | 0.47 | 0.29 |
| 16 | Benthos | 2.22 | 60.00 | 7.50 | 10.00 | 0.07 | 0.75 |
| 17 | Producers | 1.00 | 120.0 | 75.00 | - | 0.99 | - |
| 18 | Detritus | 1.00 | 80.00 | - | - | 0.12 | - |

Table S1. Ecopath model parameters

|  | Prey \ Predator | 1 | 2 | 3 | 4 | 5 | 6 | 7 | 8 | 9 | 10 | 11 | 12 | 13 | 14 | 15 | 16 |
| --- | --- | --- | --- | --- | --- | --- | --- | --- | --- | --- | --- | --- | --- | --- | --- | --- | --- |
| 1 | Large rorquals | 0 | 0 | 0.0025 | 0 | 0 | 0 | 0 | 0 | 0 | 0 | 0 | 0 | 0 | 0 | 0 | 0 |
| 2 | Small rorquals | 0 | 0 | 0.005 | 0 | 0 | 0 | 0 | 0 | 0 | 0 | 0 | 0 | 0 | 0 | 0 | 0 |
| 3 | Odontocetes | 0 | 0 | 0.005 | 0 | 0 | 0 | 0 | 0 | 0 | 0 | 0 | 0 | 0 | 0 | 0 | 0 |
| 4 | Pinnipeds | 0 | 0 | 0.05 | 0 | 0 | 0 | 0 | 0 | 0 | 0 | 0 | 0 | 0 | 0 | 0 | 0 |
| 5 | Penguins | 0 | 0 | 0.075 | 0.01 | 0 | 0.005 | 0 | 0 | 0 | 0 | 0 | 0 | 0 | 0 | 0 | 0 |
| 6 | Flying seabirds | 0 | 0 | 0.001 | 0 | 0 | 0.005 | 0 | 0 | 0 | 0 | 0 | 0 | 0 | 0 | 0 | 0 |
| 7 | Large demersals | 0 | 0 | 0.15 | 0.04 | 0 | 0.02 | 0.01 | 0 | 0 | 0 | 0 | 0 | 0 | 0 | 0 | 0 |
| 8 | Small demersals | 0 | 0 | 0.125 | 0.15 | 0.05 | 0.03 | 0.26 | 0.02 | 0 | 0.055 | 0 | 0 | 0 | 0 | 0 | 0 |
| 9 | Small pelagics | 0.05 | 0.1 | 0.25 | 0.25 | 0.325 | 0.35 | 0.11 | 0.03 | 0 | 0.225 | 0 | 0 | 0 | 0 | 0 | 0 |
| 10 | Cephalopods | 0.02 | 0.02 | 0.321 | 0.2 | 0.125 | 0.15 | 0.03 | 0.05 | 0 | 0.2 | 0 | 0 | 0 | 0 | 0 | 0 |
| 11 | Carnivorous zooplankton | 0.02 | 0.02 | 0.001 | 0 | 0 | 0.01 | 0.05 | 0.19 | 0.4 | 0.05 | 0 | 0.025 | 0 | 0 | 0 | 0.01 |
| 12 | Krill | 0.8 | 0.8 | 0.015 | 0.35 | 0.5 | 0.4 | 0.21 | 0.25 | 0.16 | 0.05 | 0 | 0 | 0 | 0 | 0 | 0.01 |
| 13 | Salps | 0.01 | 0.01 | 0 | 0 | 0 | 0.01 | 0.02 | 0.02 | 0.04 | 0.25 | 0.05 | 0 | 0 | 0 | 0 | 0 |
| 14 | Copepods | 0.1 | 0.05 | 0 | 0 | 0 | 0.02 | 0.05 | 0.19 | 0.4 | 0.02 | 0.65 | 0.025 | 0.05 | 0 | 0 | 0.01 |
| 15 | Microzooplankton | 0 | 0 | 0 | 0 | 0 | 0 | 0 | 0 | 0 | 0 | 0.1 | 0.35 | 0.15 | 0.35 | 0 | 0.15 |
| 16 | Benthos | 0 | 0 | 0 | 0 | 0 | 0 | 0.26 | 0.25 | 0 | 0.15 | 0.1 | 0 | 0 | 0 | 0 | 0.02 |
| 17 | Producers | 0 | 0 | 0 | 0 | 0 | 0 | 0 | 0 | 0 | 0 | 0 | 0.5 | 0.7 | 0.63 | 1 | 0.6 |
| 18 | Detritus | 0 | 0 | 0 | 0 | 0 | 0 | 0 | 0 | 0 | 0 | 0.1 | 0.1 | 0.1 | 0.02 | 0 | 0.2 |

Table S2. Ecopath model diet composition matrix

| Prey \ Predator | 1 | 2 | 3 | 4 | 5 | 6 | 7 | 8 | 9 | 10 | 11 | 12 | 13 | 14 | 15 | 16 |
| --- | --- | --- | --- | --- | --- | --- | --- | --- | --- | --- | --- | --- | --- | --- | --- | --- |
| Large rorquals |  |  | 3.0 |  |  |  |  |  |  |  |  |  |  |  |  |  |
| Small rorquals |  |  | 3.0 |  |  |  |  |  |  |  |  |  |  |  |  |  |
| Odontocetes |  |  | 3.0 |  |  |  |  |  |  |  |  |  |  |  |  |  |
| Pinnipeds |  |  | 2.0 |  |  |  |  |  |  |  |  |  |  |  |  |  |
| Penguins |  |  | 2.0 | 2.0 |  | 2.0 |  |  |  |  |  |  |  |  |  |  |
| Flying seabirds |  |  | 2.0 |  |  | 2.0 |  |  |  |  |  |  |  |  |  |  |
| Large demersals |  |  | 3.0 | 3.0 |  | 3.0 | 3.0 |  |  |  |  |  |  |  |  |  |
| Small demersals |  | 4.0 | 3.0 | 3.0 | 3.0 | 3.0 | 3.0 | 3.0 |  | 3.0 |  |  |  |  |  |  |
| Small pelagics | 4.0 | 4.0 | 4.0 | 4.0 | 4.0 | 4.0 | 4.0 | 4.0 |  | 4.0 |  |  |  |  |  |  |
| Cephalopods | 4.0 | 4.0 | 4.0 | 4.0 | 4.0 | 4.0 | 4.0 | 4.0 |  | 4.0 |  |  |  |  |  |  |
| Carnivorous zooplankton | 4.0 | 4.0 | 4.0 |  |  | 4.0 | 4.0 | 4.0 | 4.0 | 4.0 |  | 4.0 |  |  |  | 4.0 |
| Krill | 4.0 | 4.0 | 4.0 | 4.0 | 4.0 | 4.0 | 4.0 | 4.0 | 4.0 | 4.0 |  |  |  |  |  | 4.0 |
| Salps | 4.0 | 4.0 |  |  |  | 4.0 | 4.0 | 4.0 | 4.0 | 4.0 | 4.0 |  |  |  |  |  |
| Copepods | 4.0 | 4.0 |  |  |  | 4.0 | 4.0 | 4.0 | 4.0 | 4.0 | 4.0 | 4.0 | 4.0 |  |  | 4.0 |
| Microzooplankton |  |  |  |  |  |  |  |  |  | 2.0 | 2.0 | 2.0 | 2.0 | 2.0 |  | 2.0 |
| Benthos |  |  |  |  |  |  | 2.0 | 2.0 |  | 2.0 | 2.0 |  |  |  |  | 2.0 |
| Producers |  |  |  |  |  |  |  |  |  |  |  | 2.0 | 2.0 | 2.0 | 2.0 | 2.0 |
| Detritus |  |  |  |  |  |  |  |  |  |  | 2.0 | 2.0 | 2.0 | 2.0 |  | 2.0 |

Table S3. Ecosim vulnerability matrix

| Species | Historical abundance (000s, present study) | Historical abundance  (000s, Christensen [34]) | Current depletion level  (%, present study) | Current depletion level  (%, Christensen [34]) |
| --- | --- | --- | --- | --- |
| Blue | 311 | 327 | 99 | 99.6 |
| Fin | 429 | 625 | 99 | 96 |
| Sei | 167 | 167 | 68 | 84 |
| Humpback | 135 | 199 | 56 | 89 |
| Minke | 778 | 379 | 3 | 16 |

Table S4. Estimates of the pre-whaling abundances and current depletion levels of Antarctic rorqual populations based on this study and the analysis by Christensen [34].
